# Global education and training in geriatrics: mapping transnational initiatives and their complementarities

**DOI:** 10.1007/s41999-026-01418-w

**Published:** 2026-02-03

**Authors:** Román Romero-Ortuño, Hidenori Arai, Prasert Assantachai, José Alberto Avila Funes, Rosette Farrugia-Bonello, Siobhan Casey, Liang-Kung Chen, Gary Cheung, Jugdeep Dhesi, Fiona Ecarnot, Leon Flicker, Tamàs Fülöp, Ashish Goel, Adam L. Gordon, Radhouane Gouiaa, Celia L. Gregson, Luis Miguel Gutiérrez Robledo, José Ricardo Jauregui, Marina Kotsani, Jūratė Macijauskienė, Stefania Maggi, Finbarr C. Martin, Tahir Masud, Reshma A. Merchant, Jean-Pierre Michel, Manuel Montero-Odasso, Patricia Morsch, Thomas Münzer, Balakrishnan Kichu Nair, José F. Parodi, Grace M. E. Pearson, Mirko Petrovic, Karolina Piotrowicz, Regina Roller-Wirnsberger, Cornel C. Sieber, Gregor Sneddon, Maw Pin Tan, Nathalie van der Velde, Rohan Wee, Michael Vassallo, M. Cristina Polidori

**Affiliations:** 1 European Geriatric Medicine Society (EuGMS); Discipline of Medical Gerontology, School of Medicine, and Global Brain Health Institute, Trinity College Dublin, Dublin, Ireland; 2 National Center for Geriatrics and Gerontology, Obu, Japan; 3 Southeast Asian Academy in Geriatrics; Faculty of Medicine Siriraj Hospital, Mahidol University, Bangkok, Thailand; 4 Instituto Nacional de Ciencias Médicas y Nutrición Salvador Zubirán, Mexico City, Mexico; 5 International Institute on Ageing, United Nations-Malta (INIA), Valletta, Malta; 6 International Federation on Ageing (IFA); Commissioner for Older People for Northern Ireland, Belfast, UK; 7 Global Brain Health Institute, Trinity College Dublin, Dublin, Ireland; 8 Center for Geriatrics and Gerontology, Taipei Veterans General Hospital, Taipei, Taiwan; 9 Center for Healthy Longevity and Aging Sciences, National Yang Ming Chiao Tung University, Taipei, Taiwan; 10 Taipei Municipal Gan-Dau Hospital, Taipei, Taiwan; 11 Department of Psychological Medicine, School of Medicine, Faculty of Medical and Health Sciences, The University of Auckland, Auckland, New Zealand; 12 British Geriatrics Society; School of Life Course and Population Sciences, King’s College, London, UK; 13 Department of Cardiology, University Hospital Besançon, Besançon, France; 14 SINERGIES Laboratory, University Marie and Louis Pasteur, Besançon, France; 15 Western Australian Centre for Health and Ageing, Medical School, University of Western Australia, Perth, Australia; 16 Faculty of Medicine and Health Sciences, Research Center on Aging, Université de Sherbrooke, Sherbrooke, QC Canada; 17 Department of Medicine, AIMS, Mohali, India; 18 Academic Centre for Healthy Ageing, Barts Health NHS Trust, Whipps Cross Hospital, London, UK; 19 Wolfson Institute of Population Health, Queen Mary University of London, London, UK; 20 Policlinique CNSS, Sfax, Tunisia; 21 Global Health and Ageing Research Unit, Bristol Medical School, University of Bristol, Bristol, UK; 22 The Health Research Unit of Zimbabwe (THRU ZIM), The Biomedical Research and Training Institute, Harare, Zimbabwe; 23 WHO Collaborating Centre on Integrated Care for Healthy Aging at the Instituto Nacional de Geriatría, Mexico City, Mexico; 24 International Association of Gerontology and Geriatrics (IAGG);, Buenos Aires University and La Matanza University, Buenos Aires, Argentina; 25 PROGRAMMING COST Action 21122 and Global Europe Initiative (GEI) Group of the European Geriatric Medicine Society (EuGMS);, Hellenic Society for the Study and Research of Ageing, Athens, Greece; 26 European Union of Medical Specialists, Geriatric Medicine Section (UEMS-GMS);, Lithuanian University of Health Sciences, Kaunas, Lithuania; 27 European Interdisciplinary Council on Ageing (EICA); CNR Ageing Branch, Neuroscience Institute, Padova, Italy; 28 Population Health Sciences, King’s College London, London, UK; 29 European Geriatric Medicine Society (EuGMS);, Nottingham University Hospitals NHS Trust, Nottingham, UK; 30 Yong Loo Lin School of Medicine, National University of Singapore, Singapore, Singapore; 31 National University Hospital, Singapore, Singapore; 32 University of Geneva, Geneva, Switzerland; 33 Gait and Brain Lab, Parkwood Institute and Lawson Health Research Institute, Schulich School of Medicine and Dentistry, Department of Medicine and Division of Geriatric Medicine, and Department of Epidemiology and Biostatistics, University of Western Ontario, London, ON Canada; 34 Pan American Health Organization (PAHO)/World Health Organization (WHO), Washington, DC, USA; 35 European Academy for Medicine of Ageing (EAMA);, University of Zurich, Zurich, Switzerland; 36 School of Medicine and Public Health (Medical Education and Professional Development), The University of Newcastle, Newcastle, Australia; 37 Facultad de Medicina, Centro de Investigación del Envejecimiento, Universidad de San Martín de Porres, Lima, Perú; 38 Global Health and Ageing Research Unit, University of Bristol Medical School, Bristol, United Kingdom; 39 Older Persons Unit, Royal United Hospitals Bath NHS Foundation Trust, Bath, UK; 40 European Geriatric Medicine Society (EuGMS); Department of Internal Medicine and Paediatrics, Ghent University, Ghent, Belgium; 41 European Geriatric Medicine Society (EuGMS); Department of Internal Medicine and Gerontology , Jagiellonian University Medical College, Kraków, Poland; 42 Department of Internal Medicine, Medical University of Graz, Graz, Austria; 43 European Interdisciplinary Council on Ageing (EICA); Institute for Biomedicine of Ageing, Friedrich-Alexander-Universität Erlangen-Nürnberg, Nuremberg, Germany; 44 Department of Medicine, Kantonsspital Winterthur, Winterthur, Switzerland; 45 International Federation on Ageing (IFA), Toronto, Canada; 46 Division of Geriatric Medicine, Department of Medicine, Faculty of Medicine, Universiti Malaya, Kuala Lumpur, Malaysia; 47 European Geriatric Medicine Society (EuGMS); Amsterdam UMC, Department of Internal Medicine, Section of Geriatric Medicine, University of Amsterdam, Amsterdam, The Netherlands; 48 Amsterdam Public Health Research Institute, Amsterdam, The Netherlands; 49 Victorian Geriatric Medicine Training Program (VGMTP); Royal Australasian College of Physicians (RACP); Australian and New Zealand Society for Geriatric Medicine (ANZSGM);, St Vincent’s Hospital Melbourne, Melbourne, Australia; 50 European Geriatric Medicine Society (EuGMS); European Union of Medical Specialists, Geriatric Medicine Section (UEMS-GMS);, University Hospitals Dorset, Dorset, UK; 51 European Geriatric Medicine Society (EuGMS); Ageing Clinical Research, Department II of Internal Medicine and Centre for Molecular Medicine Cologne, Faculty of Medicine and University Hospital Cologne, University of Cologne, Cologne, Germany; 52 Excellence Cluster CECAD, University of Cologne, Cologne, Germany

**Keywords:** Geriatrics education, Education, Medical, Continuing, Health workforce, International cooperation, Capacity building, Global health

## Abstract

**Purpose:**

To map and characterise major transnational initiatives in geriatrics education and training, and explore complementarities as a basis for a more integrated and equitable global framework.

**Methods:**

A mapping exercise and expert consultation were undertaken by the European Geriatric Medicine Society (EuGMS) Special Interest Group on Education and Training between January and October 2025, including a meeting of international experts during the Twenty-First EuGMS Congress in Reykjavík. Eligible initiatives operated across national borders with an explicit mandate in education and training related to geriatrics and were not confined to a specific topic or subspecialty. Each initiative was profiled by scope, target audience, and contributions, and classified within a three-tier framework: (1) foundational capacity-building, (2) professional and interprofessional development, and (3) leadership and specialist advancement.

**Results:**

Seventeen initiatives were identified. Tier 1 included the International Federation on Ageing (IFA), International Institute on Ageing, United Nations–Malta (INIA), PAHO’s ACAPEM (Basic), ASEAN’s Centre for Active Ageing and Innovation (ASEAN–ACAI), IAGG’s e-Training in Gerontology and Geriatrics (e-TRIGGER) programmes, WHO’s Integrated Care for Older People (WHO ICOPE approach), and AfriAGE. Tier 2 included the IAGG, EuGMS, EICA, PROGRAMMING CA2112, Victorian Geriatric Medicine Training Programme (VGMTP), and ACAPEM (Intermediate); and Tier 3 was represented by leadership academies (EAMA, ALMA, MEAMA/MENAAA, and AAMA), and UEMS–GMS.

**Conclusion:**

Collectively, these programmes form a considerably disjointed but potentially complementary global ecosystem for geriatrics education. Greater mutual awareness and alignment, anchored in equity and interprofessional inclusion, could enhance efficiency and sustainability in developing the global geriatrics workforce.

**Supplementary Information:**

The online version contains supplementary material available at 10.1007/s41999-026-01418-w.

## Key summary points

**Aim:**

To map and characterise major transnational initiatives in education and training in geriatrics, and to explore complementarities to support a more coherent and equitable global framework.

**Findings:**

Multiple transnational programmes operate across a wide spectrum of structures, educational approaches, and content, reflecting diverse regional priorities and stages of development.

**Message:**

Coordinated collaboration amongst initiatives is essential to build global capacity, promote equity, and ensure sustainability in geriatrics education and workforce development.

**Supplementary Information:**

The online version contains supplementary material available at 10.1007/s41999-026-01418-w.

## Introduction

The ageing demographic shift has been accompanied by a substantial increase in geriatrics research outputs in recent years, driven by the need to translate scientific advances into practical interventions for older populations [1, 2]. Geriatric medicine typically adopts a person-centred, comprehensive approach that extends beyond traditional disease management and curative care by integrating functional, psychosocial, and preventive dimensions to support holistic well-being in later life [3]. Integrating this paradigm shift into curricula and meeting these challenges requires not only an expanded, multidisciplinary workforce but also one equipped with robust, evidence-based competencies in geriatric care from the earliest stages of education and training [4, 5]. This aligns with the need to promote integrated, person-centred health and care for older people as one of the four World Health Organization (WHO) action areas in support of the United Nations (UN) Decade of Healthy Ageing (2021–2030) [6].

In response to these demographic and workforce pressures, structured education in geriatric medicine has progressively evolved over the past decades. In Europe, the establishment of the British Geriatrics Society (BGS) in 1947 marked a seminal step in formalising training in geriatrics [7–9]. Through initiatives spanning undergraduate and postgraduate curricula, an education and training committee, a diploma programme, e-learning and microlearning resources, and an online educational hub [10], the BGS continues to influence and support the delivery of education and training in geriatric medicine across various career stages and amongst multidisciplinary professionals working with older people within the United Kingdom, whilst also being accessible internationally and inspiring the development of similar national initiatives elsewhere. Comparable national responses have since emerged in other regions, reflecting shared workforce challenges and policy priorities. For example, the Australian and New Zealand Society for Geriatric Medicine (ANZSGM) issued an Education in Geriatric Medicine position statement in 2022, asserting that geriatric education should be mandatory for all medical graduates and delivered through defined competencies in knowledge, skills, and attitudes supported by formal curricula and clinical experience [11].

As the international mobility of health professionals increases and workforce challenges become increasingly shared across health systems, these national efforts highlight the growing importance of transnational initiatives that operate across borders to strengthen global capacity in geriatrics education. A pioneering example was the International Institute on Ageing, United Nations–Malta (INIA), established in 1987 through an agreement between the United Nations and the Government of Malta. INIA was the first international institute dedicated to education and training in ageing, created to meet the needs of developing countries and bridge regional disparities [12, 13]. Since then, multiple transnational initiatives have emerged, initially in countries with ageing populations and subsequently in countries with emerging older populations. However, the landscape remains fragmented, coordination and mutual awareness are limited, levels of training vary, and approaches differ widely.

Against this backdrop of growing need and persistent fragmentation, this paper sought to identify existing gaps through a descriptive mapping of transnational initiatives in geriatrics education and training. It focussed on major international programmes that are broad in scope rather than topic-specific and that operate across national borders under regional or global coordination. The analysis used a unified, tiered framework to clarify how activities intersect across different levels of education, identify areas of overlap and unmet need, and highlight potential complementarities. Overall, this exercise aimed to inform the development of a more coherent, equitable, and sustainable global strategy for geriatrics education and workforce development, responsive to the demands of a rapidly ageing world.

## Methods

This descriptive mapping and expert consultation was led by the European Geriatric Medicine Society (EuGMS) Special Interest Group (SIG) on Education and Training, in collaboration with partner networks active in geriatrics education and training worldwide. The initiative was aligned with preparations for the Twenty-First EuGMS Congress in Reykjavík, where a dedicated session, *Global Education and Training Initiatives in Geriatrics* (26 September 2025), brought together experts from international organisations, regional academies, and professional societies to consolidate dialogue and advance a coordinated global framework for education and training in geriatrics (Supplementary Fig. 1).

Information on transnational initiatives was collected through a structured, expert-informed process conducted between January and October 2025. Data sources included official organisational websites, public reports, peer-reviewed and grey literature, and direct input from initiative leads or representatives. Initiatives were eligible to be included in this mapping exercise if: they operated across national borders with a defined regional or global mandate; focussed explicitly on education, training, or capacity-building in geriatrics and/or ageing; were not confined to a single topic or subspecialty; and were coordinated by a professional society, academic network, or intergovernmental organisation. National or single-institution programmes were excluded unless they hosted or coordinated transnational activities.

The analytical approach combined descriptive and conceptual synthesis. Initiatives were examined in relation to their origins, with attention to their chronological development, as well as their target audiences, scope, distinctive contributions, and potential alignments with other programmes. From this, a conceptual framework was developed to describe the continuum of learning and professional development across three progressive tiers:

1. Tier 1: foundational capacity-building

This tier comprises broad, introductory education and training initiatives aimed at developing essential competencies in ageing and the care of older adults. Programmes at this level typically target multidisciplinary or frontline workforces and emphasise awareness, basic skills, and system-level capacity strengthening. They are often linked to regional or global policy organisations and may use open-access or scalable learning platforms designed to reach diverse professional groups, including those in low- and middle-income settings.

2. Tier 2: professional and interprofessional development

This tier comprises structured educational initiatives aimed at advancing clinical, educational, and research competencies amongst health and social care professionals. Programmes in this category promote interprofessional learning, collaborative practice, and the harmonisation of curricula or training standards across disciplines and countries. They often include postgraduate training activities, continuing professional development, and regional networks that enhance professional mobility and quality improvement.

3. Tier 3: leadership and specialist advancement

This tier focuses on advanced programmes designed to develop academic, institutional, and policy leadership, as well as formal specialist accreditation within geriatrics and ageing-related fields. These initiatives typically include faculty-development academies, or specialist curricula and/or examinations. They aim to cultivate expert educators, researchers, and clinical leaders capable of shaping educational standards, influencing policy, and sustaining capacity-building efforts globally.

## Results

**Fig. 1:**
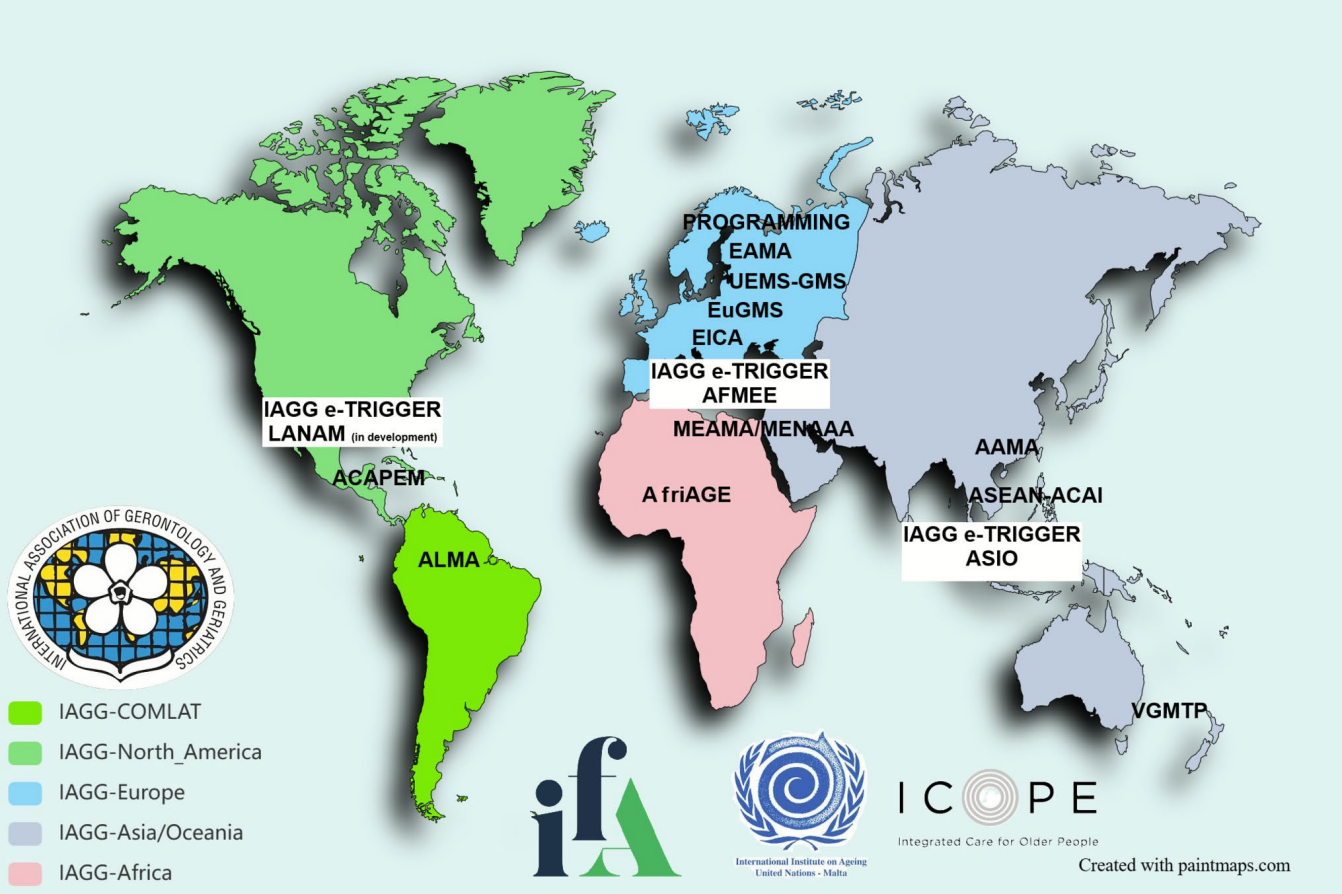
Global distribution of transnational initiatives in geriatric education and training. The map illustrates key international and regional organisations contributing to geriatrics education and capacity-building across continents. Initiatives are grouped by International Association of Gerontology and Geriatrics (IAGG) regions: IAGG–Europe (blue), IAGG–Asia/Oceania (purple), IAGG–Africa (pink), IAGG–COMLAT (green), and IAGG–North America (darker green). Programmes include leadership academies (EAMA, AAMA, ALMA, and MEAMA/MENAAA), professional organisations (EuGMS, UEMS-GMS, PROGRAMMING COST Action, and EICA), digital and blended learning platforms (ACAPEM, e-TRIGGER, and AfriAGE), and global frameworks (INIA, ICOPE approach, and IFA)

The mapping identified a diverse and interconnected landscape of transnational initiatives with direct relevance to education and training in geriatrics (Fig. 1). Each programme has distinct objectives, organisational structures, and delivery models that operate across different levels of learning and professional development. Table 1 summarises the main features, core activities, and potential complementarities of these initiatives. Table 2 outlines the three-tier classification of educational activities identified across the mapped initiatives. The text below provides a narrative summary of each initiative, accompanied by relevant references and descriptive details.

**Table 1:** Key transnational initiatives in education and training in geriatrics: unique features, contributions, and potential complementarities (ordered by year of establishment)

Initiative	Year established	Unique feature(s)	Primary contribution /key content	Potential complementarities
IAGG—International Association of Gerontology and Geriatrics Sector: Global federation of professional and academic societies Target audience: UG, PG, PR (multidisciplinary, global reach) Interdisciplinary: Yes Website: https://iagg.site/	1950 (founded in Liège, Belgium; regional structure progressively expanded between 1960 and 2009)	World’s oldest and largest multidisciplinary organisation devoted to ageing; operates through five regional divisions—Europe (1960), North America (1960), Asia/Oceania (1978), Latin America (COMLAT, 1991), and Africa (2009)	Coordinates a World Congress every four years; fosters research, education, and training in gerontology and geriatrics; provides a platform for cross-regional collaboration and policy dialogue; maintains formal partnerships with the UN and WHO; and supports education and capacity-building through the ICSO, FGE, and e-TRIGGER programmes	Serves as an umbrella for regional and thematic academies; strengthens integration amongst continental academies and professional societies; aligns global policy, education, and clinical practice; and demonstrates strong potential complementarity with IFA, INIA, EuGMS ECGI, and WHO ICOPE in advancing education, mentorship, active ageing, and integrated care frameworks
IFA – International Federation on Ageing Sector: Global NGO (policy and civil society) Target audience: PR (policymakers, NGOs, professionals) Interdisciplinary: Yes (policy–practice convenor) Website: https://ifa.ngo	1973 (headquartered in Toronto)	Global NGO integrating policy, advocacy, knowledge translation, and multi-stakeholder convening; convenes Global Conferences on Ageing since 1992 on an approximately biennial basis	Integrates evidence, policy and practice through frameworks, webinars and advocacy resources that influence national and global ageing agendas; operates as a recognised UN ECOSOC and WHO partner platform	Complements INIA and WHO ICOPE by embedding gerontology and geriatrics education within broader frameworks for healthy and age-inclusive ageing
INIA – International Institute on Ageing, United Nations–Malta Sector: UN-affiliated institute (education, research, policy capacity-building) Target audience: PR (policymakers, professionals, NGOs, educators) Interdisciplinary: Yes (global and cross-sector training) Website: https://www.inia.org.mt	1987 (established through an agreement between the United Nations and the Government of Malta)	UN institute dedicated to training, research, and policy development in ageing; acts as a bridge between LMIC and high-income countries	Undertakes training programmes tailored to the needs of developing countries; promotes technical cooperation, research, and publication of educational materials; supports the implementation of the Vienna (1982) and Madrid (2002) International Plans of Action on Ageing; and provides advisory services to governments and organisations	Complements WHO ICOPE and IFA by strengthening foundational and system-level capacity for healthy and active ageing; supports IAGG’s education and training objectives through applied policy, workforce development, and global cooperation
EAMA – European Academy for Medicine of Ageing Sector: Advanced postgraduate training programme (academic and clinical leadership in geriatrics) Target audience: PG, PR (junior medical faculty, future educators, clinical leaders) Interdisciplinary: No (primarily medical and clinical faculty) Website: https://www.eama.eu	1995 (founded as an advanced postgraduate course in geriatric medicine)	Two-year postgraduate programme comprising four one-week residential sessions held twice annually (January and June) across Europe; designed for emerging academic and clinical leaders in geriatrics	Emphasises leadership, teaching, research, and management competencies; guided by a structured catalogue of learning objectives covering knowledge, skills, and attitudes; promotes interactive, peer-based learning, mentoring, and networking; conducted in English	Complements other regional leadership academies, EuGMS ECGI, and IAGG ICSO by advancing leadership and faculty development in geriatric education and promoting academic excellence in ageing medicine
UEMS-GMS – European Union of Medical Specialists, Geriatric Medicine Section Sector: European professional body (specialist accreditation and postgraduate training standards) Target audience: PR (specialist trainees, trainers, accreditation bodies, academic institutions) Interdisciplinary: No (medical specialty focussed) Website: https://www.uemsgeriatricmedicine.org	1997 (inaugurated as a specialist section within the UEMS)	Defines and updates the ETR for geriatric medicine; establishes common postgraduate training standards across Europe; co-organises the EGeMSE with the BGS and the Federation of Royal Colleges of Physicians of the UK	Develops and maintains the ETR (latest 2025 version) structured around trainees, trainers, and training institutions; integrates Entrustable Professional Activities (EPAs) and competency-based assessment; contributes to the European Undergraduate Curriculum in Geriatric Medicine (2014; currently being updated); collaborates with other UEMS sections to embed ageing and geriatric care across medical education	Complements EuGMS, EAMA, EICA, and IAGG by aligning postgraduate standards and examinations with competency-based education; reinforces WHO ICOPE and the UN Decade of Healthy Ageing (2021–2030) through harmonised training and quality-of-care standards
EuGMS – European Geriatric Medicine Society Sector: European professional society (clinical, research, education, and policy integration) Target audience: PR, PG (clinicians, educators, researchers, early-career professionals) Interdisciplinary: Yes Website: https://www.eugms.org	2000	European professional society promoting geriatric medicine, education, and healthy ageing across Europe; delivers an annual EuGMS Congress as a major platform for scientific exchange, education, and policy dialogue; coordinates multiple Special Interest Groups (SIGs), including Education and Training and the Early-Career Geriatricians Initiative (ECGI)	Advances education and training through the Education and Training SIG and ECGI; delivers a Core Curriculum vertical strand at the annual congress in collaboration with EAMA, ECGI and GEI; and, through the Clinical Guidelines Committee, develops evidence-based recommendations, including care-home guidelines and competency frameworks. Also hosts the PROGRAMMING COST Action 21122 and maintains international collaborations	Complements UEMS-GMS, EAMA, EICA, and IAGG by aligning postgraduate and continuing education with harmonised standards; reinforces WHO ICOPE and the UN Decade of Healthy Ageing (2021–2030) through shared objectives in workforce development, clinical quality, and knowledge translation
ALMA – Latin American Academy of Medicine of Ageing (Academia Latinoamericana de Medicina del Adulto Mayor) Sector: Regional postgraduate training academy (education, research, and policy advancement) Target audience: PG, PR (junior faculty, clinicians, educators, and geriatric specialists) Interdisciplinary: No (primarily medical and clinical faculty) Website: https://www.almageriatria.org	2002	Nonprofit regional organisation promoting excellence in geriatric medicine, education, and ageing policy across Latin America; functions as an advanced postgraduate programme for emerging academic and clinical leaders	Structured as a four-session advanced postgraduate course with 1-week residential modules hosted in various Latin American and Hispanic locations; delivers the recurring *Curso ALMA*, research seminars, publications, and—with PAHO—virtual learning for primary care professionals. Maintains an alumni network spanning Latin America, the Caribbean, Spain, and communities in the U.S.	Complements other leadership academies, IAGG, and PAHO/WHO ICOPE by strengthening postgraduate and system-level training in geriatric medicine
MEAMA – Middle East Academy for Medicine of Ageing and MENAAA – Middle East and North Africa Association on Aging and Alzheimer’s Sector: Regional postgraduate training academy and multidisciplinary network (education, research, and policy) Target audience: PG, PR (junior faculty, clinicians, educators, allied health professionals, policymakers) Interdisciplinary: Yes (medical, allied health, and policy collaboration) Website: https://menaaa.org	2002 (MEAMA); 2014 (MENAAA)	MEAMA – regional postgraduate academy modelled on the European Academy for Medicine of Ageing (EAMA); provides 2-year advanced courses with four 1-week residential sessions; has trained over 600 professionals from across the Middle East. MENAAA – umbrella association integrating education, research, and policy on ageing and Alzheimer’s across the MENA region	Delivers advanced training modules combining lectures, interactive seminars, and group projects; promotes regional networks of geriatricians and educators; collaborates with the INIA and MENAAA; jointly offers the Diploma in Dementia Care, a four-session, ten-module programme for multidisciplinary professionals	Complements all leadership academies, IAGG, and WHO ICOPE by strengthening postgraduate and system-level training in geriatric medicine and dementia care
VGMTP – Victorian Geriatric Medicine Training Programme Sector: Regional specialist training programme (Australia–New Zealand framework) Target audience: PG, PR (advanced trainees, junior doctors, nurses, and allied health professionals) Interdisciplinary: Yes Website: https://www.vgmtp.org	2005	Operates within the transnational framework of the RACP, which governs specialist training across Australia and New Zealand; serves as a key regional hub for geriatric education	Delivers two-to-three online seminars or lectures each month within a 2-year curriculum cycle; provides structured training for Advanced Trainees in Geriatric Medicine; offers free online education modules and resources for non-specialists, junior doctors, and nurses; supports mentorship and professional development to strengthen geriatric care across the Australasian region	Complements IAGG, EuGMS, and WHO ICOPE by strengthening regional capacity for specialist and multidisciplinary training; supports alignment between national postgraduate curricula and global competency-based education in geriatric medicine
AAMA – Asian Academy for Medicine of Ageing Sector: Regional postgraduate training academy (education, leadership, and clinical development) Target audience: PG, PR (junior faculty, clinicians, educators, and geriatric specialists) Interdisciplinary: Yes Website: N/A	2011	Regional postgraduate academy for education and leadership in geriatric medicine and ageing; structured around the EAMA “teach-the-teachers” model; promotes regional collaboration and capacity-building across Asia	Conducts annual advanced education and training programmes, notably the IAGG Master Class on Ageing in Asia, held 13 times as of 2025; strengthens capacity in ageing research, clinical geriatrics, and leadership development; fosters regional academic and professional networks	Complements all leadership academies, IAGG, and WHO ICOPE by advancing postgraduate and leadership training in geriatric medicine; supports alignment of Asian regional capacity-building with global frameworks for education and healthy ageing
EICA – European Interdisciplinary Council on Ageing Sector: European multidisciplinary educational and translational council (education, research, and policy integration) Target audience: UG, PG, PR (students, early-career researchers, clinicians, educators, policymakers) Interdisciplinary: Yes (multisectoral and cross-disciplinary) Website: https://eica.univiu.org	2015	European multidisciplinary platform translating ageing research into education and policy; awards EICA Certificates to institutions demonstrating excellence in integrating ageing, health, and care knowledge	Organises interdisciplinary courses, seminars, and workshops in collaboration with VIU, EuGMS, IAGG, and IFA; delivers the annual Interdisciplinary Summer Institute on Ageing (12th edition in 2025) for students and early-career professionals; promotes evidence translation, leadership, and collaboration across research, policy, and care sectors	Complements EuGMS, IAGG, and IFA by bridging research, education, and policy; the Summer Institute synergises with IAGG ICSO and EuGMS ECGI by advancing early-career mentorship, interdisciplinary learning, and international knowledge exchange
ACAPEM – Development of Competences in Health Care for Older Persons Sector: Regional virtual learning and capacity-building programme (education and workforce development) Target audience: PG, PR (primary care professionals, nurses, physicians, allied health workers, policymakers) Interdisciplinary: Yes Websites: Basic Level: https://campus.paho.org/en/course/health-care-older-persons-acapem-basic-level; Intermediate Level: https://campus.paho.org/en/course/ACAPEM-Intermediate-Level	2017	Regional PAHO/WHO initiative providing competency-based training in healthy ageing and older-adult care through three progressive online levels (Basic, Intermediate, and forthcoming Advanced); delivered in English, Spanish, and Portuguese via the PAHO Virtual Campus for Public Health	Equips all professionals with relevant knowledge for the care of older adults; aligns with the WHO Healthy Ageing framework and the UN Decade of Healthy Ageing (2021–2030); Basic level focuses on awareness and foundational geriatric care principles, whilst the Intermediate level strengthens clinical and system competencies based on the WHO ICOPE model	Complements WHO ICOPE, INIA, IFA, by strengthening workforce competencies and advancing scalable, interprofessional training across Latin America and the Caribbean
ASEAN-ACAI Centre for Active Ageing and Innovation Sector: Regional policy, education, and innovation hub (ASEAN intergovernmental collaboration) Target audience: PR (ASEAN policymakers, health and social care professionals, educators, researchers, community leaders) Interdisciplinary: Yes (health, social care, technology, and policy sectors) Website: https://asean-acai.org	2020	ASEAN regional coordination and knowledge-sharing platform supporting active ageing and innovation across Member States	Conducts regional capacity-building workshops, executive leadership programmes, and Country Coordinators Training Workshops; delivers joint training with the National University of Singapore (NUS); organises networking dialogues and consultative meetings; supports the ASEAN Active Ageing Index	Complements WHO ICOPE, IFA, EICA, and IAGG by advancing transnational education, leadership, and innovation in ageing; bridges ASEAN member-state initiatives and promotes harmonised workforce and policy development across the region
PROGRAMMING (COST Action 21122) Sector: European Cooperation in Science and Technology (COST) Target audience: UG, PG, PR (educators, trainees, young researchers and innovators) Interdisciplinary: Yes Website: https://cost-programming.eu	2022	Pan-European network strengthening geriatrics across Europe, with particular emphasis on countries where geriatric medicine is emerging	Maps training provision and educational needs; develops consensus content; builds capacity via events, workshops, schools, and mobility grants	Synergises with EuGMS, UEMS-GMS, EAMA, EICA, and WHO ICOPE; connects national societies to European standards; promotes interprofessional education
IAGG – e-TRIGGER (e-TRaining in Gerontology and GERiatrics) Sector: Global education initiative (IAGG-FGE) Target audience: PR, PG (multidisciplinary trainers) Interdisciplinary: Yes Website: https://iagg-fge.org/ https://afmee.iagg-fge.org/ (AFMEE) https://asio.iagg-fge.org/ (ASIO)	2023 (AFMEE) 2024 (ASIO, with regional activities since 2021)	Structured 12-month training delivered through regional boards (AFMEE, ASIO, forthcoming LANAM)	Builds regional training capacity; equips educators with geriatric content and teaching skills	Connects graduates to EuGMS, EAMA, AAMA, ALMA and other leadership pipelines; aligns with WHO ICOPE and UEMS-GMS frameworks
ICOPE Training Programme approach (WHO) Sector: Global competency-based training programme (education, health systems, and workforce development) Target audience: PR, PG Interdisciplinary: Yes (multisectoral and multidisciplinary) Website: https://www.who.int/tools/icope-training-programme	2024	Global WHO-led competency-based framework with modular training, digital tools, and dissemination resources supporting the application of the ICOPE Guidelines	Anchors workforce education in evidence-based competencies for integrated, person-centred care; 19-module curriculum covering intrinsic capacity assessment, communication, ageism, social and caregiver support, and Training-of-Trainers pathway	Complements ACAPEM, INIA, and IAGG by operationalising the WHO Healthy Ageing framework; provides a universal competency framework linking regional and global standards and promoting interdisciplinary, scalable training for primary and community teams
AfriAGE (African Ageing and Geriatrics Network) online teaching programme Sector: Academic–professional collaboration network Target audience: PR, PG (multidisciplinary, Africa-wide) Interdisciplinary: Yes Website: https://globalhealthageing.blogs.bristol.ac.uk/education-and-training/	2025	Grassroots academic–clinical partnership linking African and UK institutions; low-cost, peer-led digital education model tailored for low-resource settings	Delivered the first structured Geriatric Medicine Teaching Programme (March–May 2025), a ten-week online course covering core geriatric topics	Complements IAGG-Africa, IAGG e-TRIGGER (AFMEE), and MENAAA, and WHO ICOPE by providing a flexible, scalable model for geriatric training in low- and middle-income countries

*UG* Undergraduate, *PG* Postgraduate, *PR* Practising professional, *LMIC* Low- and middle-income countries

**Table 2:** Educational tiers in transnational geriatrics education and training: scope, representative initiatives, and complementarities

Educational tier	Representative initiatives
Tier 1 – Foundational capacity building (interdisciplinary)	International Federation on Ageing International Institute on Ageing, United Nations–Malta ACAPEM—Development of Competences in Health Care for Older Persons (Pan American Health Organization) (basic) Association of Southeast Asian Nations Centre for Active Ageing and Innovation e-Training in Gerontology and Geriatrics (International Association of Gerontology and Geriatrics) World Health Organization Integrated Care for Older People training approach African Ageing and Geriatrics Network
Tier 2 – Professional and interprofessional development (interdisciplinary)	International Association of Gerontology and Geriatrics European Geriatric Medicine Society Victorian Geriatric Medicine Training Programme European Interdisciplinary Council on Ageing ACAPEM (intermediate) PROmoting GeRiAtric Medicine in countries where it is still eMergING (COST Action 21122)
Tier 3 – Leadership and specialist advancement (non-interdisciplinary)	European Academy for Medicine of Ageing European Union of Medical Specialists – Geriatric Medicine Section Latin American Academy of Medicine of Ageing Middle East Academy for Medicine of Ageing/Middle East and North Africa Association on Aging and Alzheimer’s Asian Academy for Medicine of Ageing

### International Association of Gerontology and Geriatrics (IAGG)

Founded in 1950 in Liège, Belgium [14, 15], the IAGG is the world’s oldest and largest multidisciplinary organisation dedicated to advancing excellence in gerontology and geriatrics worldwide, including in education and training [16]. The IAGG convenes a World Congress every four years [17] and operates through five regional divisions: Europe (established in 1960), North America (also in 1960, in partnership with Canadian and U.S.-based societies), Asia/Oceania (1978), Latin America (COMLAT: *Comité Latinoamericano y del Caribe*, 1991), and Africa (formally established in 2009) [18, 19]. These regional divisions collectively coordinate educational, research, and policy activities across continents. Through its Federation of Geriatric Education (FGE), the IAGG provides the overarching framework for the e-TRIGGER online training programme (see below), which involves participants across different professional domains, receiving interprofessional instruction on an integrated platform available across different continents. The IAGG also hosts the International Council of Student Organisations (ICSO), which supports undergraduate, postgraduate, and early career professionals in building a global, multidisciplinary foundation in gerontology and geriatrics [20]. In addition, IAGG maintains formal interfaces with the United Nations (UN) and the World Health Organization (WHO).

### IAGG e-TRIGGER programme (e-Training in Gerontology and Geriatrics)

The e-TRIGGER programme is a global initiative coordinated by the FGE of the IAGG. It is delivered through dedicated regional courses: ASIO (Asia and Oceania), whose first intake started in December 2021 and, at the time of writing, is soon beginning its fifth consecutive edition; AFMEE (Africa, Middle East, and Europe), whose first intake was from May 2023 to April 2024; and LANAM (Latin and North America), a third course currently under development [21, 22]. The programme runs over 12 months, offering monthly 3-h online sessions, with interactive learning activities, and certification of participation for participants who successfully achieve a pass grade on 10 out of the 12 CME quizzes organised after every monthly session. Its core aim is to build local and regional capacity by equipping trainees with up-to-date knowledge and educational methods in geriatrics and gerontology. Its target audience encompasses healthcare professionals from all backgrounds, who work with older adults in any setting. By adapting content to diverse cultural and healthcare contexts, and with its strong focus on interprofessional education, e-TRIGGER disseminates international best practices and strengthens the global network of professionals. An evaluation of the AFMEE and ASIO courses reported high satisfaction, direct application of acquired knowledge in clinical practice, improved skills in caring for older adults, and career advancement [23].

### International Federation on Ageing (IFA)

The IFA, founded in 1973 and based in Toronto, is a global non-governmental organisation working at the intersection of policy, advocacy, and ageing. Whilst not a professional society, it contributes to knowledge mobilisation and translation by convening Global Conferences on Ageing, held on an approximately biennial basis since 1992, as well as thematic workshops that serve as multidisciplinary platforms for knowledge exchange amongst academics, clinicians, policymakers, non-governmental organisations, and civil society [24]. Holding general consultative status with the United Nations Economic and Social Council and formal relations with the WHO, the IFA generates advocacy to help shape and influence policy. IFA’s outputs include, but are not limited to, policy briefs, white papers, surveys, webinars, roundtables, expert meetings, educational and communication resources, research and data findings, as well as a range of materials that support stakeholder convening (e.g., meeting summaries and consensus statements) [25]. In doing so, it complements more clinically oriented initiatives by embedding gerontology and geriatrics within global agendas on healthy ageing and age-inclusive policy.

### International Institute on Ageing, United Nations—Malta (INIA)

INIA was established in 1987 following an agreement between the UN and the Government of Malta [26]. Its main objective is to undertake training programmes that meet the needs of low- and middle-income countries and to serve as a practical bridge between them and high-income countries. Other activities include research and data collection, publication of materials, and technical cooperation. INIA supports the implementation of global ageing policy frameworks, including the Vienna International Plan of Action on Ageing (1982) [27] and the Madrid International Plan of Action on Ageing (2002) [28]. It acts as a conduit between international policy and practical capacity-building by offering training programmes, research opportunities, and advisory services for all countries. INIA collaborates with numerous international partners whilst remaining responsive to regional and national needs [29–31].

### European Academy for Medicine of Ageing (EAMA)

The EAMA, established in 1995, is an advanced postgraduate course in geriatrics for emerging academic and clinical leaders in geriatric medicine, initially in Europe [32, 33]. The 2-year programme comprises four 1-week residential sessions held twice annually (in January and June) at different European locations. It targets junior faculty and future educators in geriatrics and related disciplines. The curriculum emphasises leadership, teaching, research, and management competencies, underpinned by a comprehensive learning-objectives catalogue covering knowledge, skills, and attitudes agreed by international experts [34]. The programme promotes peer-based interactive learning, mentoring, and cross-national networking; and instruction is delivered in English. Since inception (at the time of writing), 15 postgraduate courses have been completed, drawing participants from across Europe and beyond. Through its alumni network, EAMA has contributed significantly to building clinical, educational, and research leadership in geriatric medicine in Europe and globally.

### European Union of Medical Specialists-Geriatric Medicine Section (UEMS-GMS)

The UEMS-GMS, founded in 1997 [35], underpins postgraduate training in geriatric medicine across Europe by defining and periodically updating the European Training Requirements (ETR), which establish standards for competencies, trainers, and training institutions [36]. The current 2025 ETR for the Specialty of Geriatric Medicine, developed through stakeholder consultation and expert review, builds on the 2019 European Postgraduate Curriculum in Geriatric Medicine [37] and the 2020 ETR. The final version, endorsed by EuGMS, EAMA, IAGG, and EICA [36], introduces a modernised competency-based framework structured around trainees, trainers, and institutions. It expands theoretical content, further integrates Entrustable Professional Activities (EPAs) for competence assessment, and incorporates the newly piloted European Geriatric Medicine Specialty Examination (EGeMSE) [38], a voluntary, knowledge-based exam co-organised with the BGS and the Federation of Royal Colleges of Physicians of the UK, to promote harmonised standards and professional mobility (the first pilot exam took place on 23 April 2025). Although primarily designed for EU/EEA member states, the ETR and examination framework influence geriatric medicine training internationally. The UEMS-GMS also contributed to the European Undergraduate Curriculum in Geriatric Medicine (2014) [39] (currently being updated) and continues to collaborate with other UEMS sections to strengthen the integration of ageing and geriatric care across medical disciplines. The 2025 ETR reflects the goals of the UN Decade of Healthy Ageing (2021–2030), advancing educational excellence and person-centred, integrated care for older adults whilst supporting the growth of geriatric medicine in emerging health systems.

### European Geriatric Medicine Society (EuGMS)

Founded in 2000 [40], the EuGMS promotes the development of geriatric medicine and healthy ageing across Europe by fostering excellence in clinical care, research, education, and policy. Its annual EuGMS Congress serves as a major platform for scientific exchange, professional education, and policy dialogue, attracting participants from across Europe and beyond. The Society advances education and training through multiple mechanisms, including its Special Interest Group (SIG) on Education and Training and the Early-Career Geriatricians Initiative (ECGI). The Education and Training SIG supports the Society’s mission by convening educators, sharing best practices, hosting webinars and open meetings, and developing or endorsing educational resources that strengthen national and regional capacity [41]. The ECGI, launched in 2019, is a group whose strategic mission is to engage early-career doctors (primarily in geriatric medicine, but also in internal medicine or family medicine in countries where geriatric medicine is not a recognised specialty) through structured working groups that promote networking, mentorship, leadership development, and cross-country learning opportunities, including a rotation programme and active participation in congresses and academic publications [42]. The EuGMS also delivers a Core Curriculum vertical strand at its annual congresses in collaboration with the EAMA (6 sessions), the ECGI (1 session), and the EuGMS Global Europe Initiative (GEI) (1 session), which promotes geriatric education and training in targeted countries [43]. In parallel, the Clinical Guidelines Committee aims to develop evidence-based recommendations, including a competency framework for doctors working in care homes (in collaboration with the WHO), to harmonise geriatric practice standards across Europe [44]. In addition, EuGMS hosts the PROGRAMMING COST Action 21122 (see below) and maintains international collaborations that extend its educational and policy influence globally.

### PROGRAMMING COST Action 21122 (PROmoting GeRiAtric Medicine in countries where it is still eMergING)

The PROGRAMMING COST Action 21122 (2022-2026), hosted by EuGMS and funded through the European Cooperation in Science and Technology (COST) Association, aims to strengthen geriatric medicine particularly in countries where the specialty is underdeveloped. Its core contribution is to map current training provision and educational needs, reach consensus on core educational content tailored to different professional groups and care settings, and develop methods for delivering such training effectively. The Action brings together clinicians, educators, policymakers, gerontologists, and allied experts to foster collaboration and build capacity through workshops, training schools, mobility grants, short-term scientific missions, and stakeholder engagement, and dissemination activities [45–48].

### European Interdisciplinary Council on Ageing (EICA)

The EICA, based at Venice International University (VIU, San Servolo Island, Venice), was established in 2015 with the support of the EuGMS to address the educational and translational gap between ageing research and practice [49]. Its mission is to identify interdisciplinary challenges in ageing, health, and care; to develop and implement educational strategies that promote knowledge exchange across disciplines; and to integrate scientific evidence into professional practice and policy. EICA organises interdisciplinary courses, seminars, and workshops in collaboration with VIU, EuGMS, the IAGG, and the IFA, targeting healthcare professionals, policymakers, academics, and service providers. It also develops and awards EICA Certificates to institutions that demonstrate excellence in applying comprehensive interdisciplinary knowledge on ageing, health, and care in everyday practice and policy. In addition, EICA contributes to the delivery of the annual interdisciplinary *Summer Institute on Ageing* for students and early-career researchers (12th edition in 2025) [50]. EICA promotes multidisciplinary education, fosters collaboration amongst researchers and practitioners [51–53], and advances the translation of evidence-based knowledge into improved health and social care for older adults across Europe.

### Latin American Academy of Medicine of Ageing (ALMA)

ALMA (*Academia Latinoamericana de Medicina del Adulto Mayor*) is a nonprofit organisation dedicated to promoting excellence in geriatric care and education across the Latin American region. Its mission is to serve as a regional reference point for improving the health of older adults through the advancement of geriatric training, policy analysis, and advocacy. Founded in 2002, ALMA functions primarily as an advanced postgraduate programme designed to train the next generation of academic and clinical leaders in geriatric medicine [54, 55]. Structured as a four-session course comprising intensive 1-week residential modules held in different Latin American and Hispanic locations, it targets junior faculty members and academic instructors in geriatrics. ALMA delivers a range of academic activities, including the recurring *Curso ALMA* [56], an in-person course hosted in different Latin American countries, alongside research seminars, publications, and, together with the Pan American Health Organization (PAHO), virtual learning opportunities addressed to primary care health professionals (e.g., a Course for Managers of Services for Older Adults delivered in three languages—Spanish, English, and Portuguese—with more than 350 graduates). Its alumni network extends across most of Latin America and the Caribbean islands, as well as Spain and the Hispanic colleagues in the United States, underscoring its broad regional reach.

### ACAPEM (desarrollo de competencias en la atención de salud para las personas mayores/development of competences in health care for older persons/desenvolvimento de competências para a atenção à saúde das pessoas idosas)

ACAPEM was launched in 2017 as a regional strategy led by PAHO/WHO to strengthen the competencies of health providers across Latin America and the Caribbean in the care of older people, with a particular focus on primary care. Its objective is to equip all professionals with evidence-based knowledge to deliver better care for older adults, in alignment with the WHO Healthy Ageing framework and the UN Decade of Healthy Ageing (2021–2030). The ACAPEM pathway promotes continuous learning through virtual courses hosted on the PAHO Virtual Campus for Public Health [57], structured into three progressive levels: Basic [58], Intermediate [59], launched in 2022, and a future Advanced level, each designed to progressively enhance care-related competencies. The Basic level focuses on increasing awareness of population ageing and promoting the principles of quality, person-centred care for older adults. It is recommended for all individuals, health and care providers, including community workers. The Intermediate level places emphasis on strengthening clinical and system-level knowledge for the care of older people, in alignment with the WHO ICOPE approach model. It is primarily recommended for interprofessional primary care teams and social workers, although it remains open to any professionals interested in the topic. Completing the ACAPEM-basic before enrolling in ACAPEM-Intermediate is highly encouraged. As of November 2025, the ACAPEM-Basic course, available in Spanish, English, and Portuguese, has enrolled 158,289 participants, of whom 109,659 have completed the programme. Participation is highest in Mexico (86,041 enrolled, 66,938 certified), followed by Peru, El Salvador, Ecuador, Colombia, and Chile. Most participants are women (73%), primarily aged 26–35 years (38%) and 36–45 years (24%), and work mainly in primary care centres (61%) and hospitals (28%). By profession, the largest groups are nursing professionals and general physicians, with participation also from psychologists, dentists, physiotherapists, and other health workers [60]. The ACAPEM-Intermediate course, currently available in English and Spanish with a Portuguese version under development, has enrolled 44,164 participants, of whom 30,954 have completed it. Mexico again leads participation (26,045 enrolled, 20,018 certified), followed by El Salvador, Ecuador, Chile, and Colombia. Women comprise 75% of participants, most aged 26–35 (38%), 36–45 (23%), and 18–25 (23%), with the majority employed in primary care centres (60%) and hospitals (31%) [60]. Although ACAPEM focuses on Latin America and the Caribbean, ACAPEM-Basic has engaged participants from 133 countries, and ACAPEM-Intermediate from 52 countries.

### Middle East Academy for Medicine for Ageing (MEAMA)

The MEAMA was established in 2002 [61] as a regional postgraduate training programme to advance geriatric medicine and the care of older adults across the Middle East. Modelled on the educational structure of the EAMA, MEAMA focuses on strengthening leadership, clinical expertise, and educational capacity in geriatrics [62]. Its mission is to stimulate the development of ageing-related health services, enhance clinicians’ competencies in the care of older people, and promote regional networks of geriatricians, educators, and allied health professionals. The 2-year programme consists of four intensive 1-week residential sessions combining lectures, interactive seminars, case discussions, and group projects, and has trained more than 600 health professionals from across the region to date. Whilst initially directed at physicians, MEAMA’s courses have progressively expanded to include nurses, social workers, psychologists, physiotherapists, and healthcare officers, reflecting its multidisciplinary commitment. MEAMA collaborates closely with international and regional partners, including INIA, and the Middle East and North Africa Association on Aging and Alzheimer’s (MENAAA) [63], established in 2014 to integrate research, education, and policy on ageing and dementia across the MENA region. In partnership with MENAAA, MEAMA also delivers specialised educational programmes such as the Diploma in Dementia Care [64], composed of four sessions and ten modules tailored for multi-professional participants. Through these initiatives, MEAMA contributes to capacity-building in geriatric medicine and dementia care, supports inter-regional collaboration across the Middle East, North Africa, and the broader Eastern Mediterranean, and advances the development of ageing-related health services throughout the region.

### Victorian Geriatric Medicine Training Programme (VGMTP)

The VGMTP was established in 2005 as an initiative of the ANZSGM (Victorian Division), with funding from the Victorian Department of Health [65]. Operating within the transnational framework of the Royal Australasian College of Physicians (RACP), which oversees specialist training across Australia and New Zealand, the programme aligns with the RACP curriculum in geriatric medicine [66] and functions as a key regional hub for advanced physician education. The programme delivers two-to-three online seminars or lectures each month within a two-year curriculum cycle, providing structured learning for Victorian Advanced Trainees in geriatric medicine. In addition to specialist training, the VGMTP offers free online education modules and resources aimed at non-specialists, junior doctors, nurses, and other healthcare professionals, thereby supporting broader capacity-building in the care of older adults across the Victorian and wider Australasian health systems [67].

The RACP oversees specialist training in geriatric medicine, which is a 3-year programme for doctors who have passed the Clinical Examination in Internal Medicine. Education and training activities across Australia and New Zealand are based on the RACP curriculum and are coordinated by the ANZSGM through its Geriatric Medicine Education and Training Committee, which sets policies and provides guidance across both countries. In addition to nationally coordinated activities, ANZSGM supports education through state-based branches that run training workshops and teaching programmes, as well as ongoing educational webinars. The Australian Association of Gerontology also contributes through the delivery of active education programmes.

### Asian Academy for Medicine of Ageing (AAMA)

The AAMA was established in 2011 as a regional academy modelled on the EAMA. Its mission is to strengthen education, training, and leadership in geriatric medicine and ageing across Asia. Drawing on the EAMA structure, it has conducted annual regional advanced education and training activities, notably the *IAGG Master Class on Ageing in Asia* [68], which has been held 13 times as of 2025 [69–71], to enhance capacity in ageing research and clinical geriatrics whilst fostering collaboration and leadership development across diverse health systems in the region. The Master Class is hosted by a different Asian country each year. Each annual programme spans 3 days and includes keynote lectures delivered by tutors from different countries. Group discussions are led by sub-tutors, who regularly comprise alumni of previous programmes, and are generally case-based. Each delegate is required to submit an abstract and present a poster on research they have conducted. Additional presentation opportunities are offered to nominated group representatives, who deliver case presentations. The residential programme places a strong emphasis on networking and the establishment of future collaborations amongst young leaders within Asia, as well as on mentorship from tutors and sub-tutors. The last two Master Classes have adopted an interdisciplinary approach: in 2024, participants included researchers and allied health professionals, and in 2025, a dedicated gerontology session was introduced, with tutors in gerontechnology, social gerontology, economics, and physiotherapy.

### ASEAN Centre for Active Ageing and Innovation (ACAI)

In the region of Southeast Asia, a development was the launch of the South East Asia Academy for Geriatric Medicine (SEAGM) in 2018 with the aim to strengthen geriatric training and leadership across ASEAN countries, following the EAMA-style “train-the-trainers” model. Its inaugural activity was the 9th Asian/1st Southeast Asian Master Class on Ageing, held at the Faculty of Medicine, Siriraj Hospital, Mahidol University, Bangkok, in 2018, and organised jointly with the AAMA under the auspices of the IAGG [72, 73]. The 10th Asian/2nd Southeast Asian Master Class on Ageing, held in May 2019 in the Philippines, was the final Master Class conducted to date, prior to the COVID-19 pandemic.

On the other hand, the ASEAN Centre for Active Ageing and Innovation (ACAI) was officially launched in 2019 at the 35th ASEAN Summit under Thailand’s ASEAN Chairmanship [74], and formally established in 2020 [75]. Based within the Ministry of Public Health in Nonthaburi, Thailand, ACAI serves as a regional knowledge and innovation hub to prepare ASEAN Member States for population ageing and to promote the health and well-being of older adults across Southeast Asia. Its core missions are to function as a knowledge centre on active ageing and innovation; to support evidence-informed policies, strategies, and guidelines on ageing; to implement capacity-development programmes; and to conduct research and innovation in active and healthy ageing. ACAI also facilitates cross-border collaboration amongst ASEAN Member States and international and regional partners, including WHO [76], in strengthening health and social systems for ageing populations. Through its emphasis on training, policy exchange, and research translation, ACAI provides a transnational platform for education and workforce development relevant to geriatric medicine, long-term care, and integrated care for older persons across the ASEAN region.

### WHO Integrated Care for Older People (ICOPE) Training Programme approach

The WHO ICOPE Training Programme approach was launched in 2024 [77] to strengthen workforce capacity for delivering person-centred, integrated care for older adults, in alignment with the WHO ICOPE Guidelines [78, 79]. These guidelines provide evidence-based recommendations on the community-based assessment of physical and mental capacities, intrinsic capacity, and age-related clinical syndromes, as well as interventions to prevent, slow, or reverse declines, thereby re-orienting care systems from disease-focussed management towards the maintenance of intrinsic capacity and functional ability. The training programme supports this shift by equipping health and care workers with the competencies required to disseminate ICOPE at the primary healthcare level, a cornerstone for achieving Universal Health Coverage and the UN Decade of Healthy Ageing (2021–2030).

The ICOPE Training Programmes are usually requested by Member States (i.e., national Ministries of Health [MoH]) from WHO to receive support in building the capacity of their workforce. In these activities, WHO may rely on local experts to facilitate and disseminate the training. This is also the rationale for the Training-of-Trainers module. Usually, a MoH asks WHO to conduct a 3–4 day workshop to train future local trainers in the country. At the same time, the training material is made openly available for anyone who may wish to access it.

The modular, competency-based programme is designed for multidisciplinary teams (including physicians, nurses, community health workers, physiotherapists, occupational therapists, pharmacists, psychologists, nutritionists, and social workers) and can be delivered through online and in-person formats. It comprises 19 modules that address key elements of the ICOPE approach, such as ageism, communication, care pathways, assessment of intrinsic capacity (cognition, locomotor, vitality, vision, hearing, psychological, and continence domains), age-friendly environments, social care, and caregiver support. A dedicated “Training-of-Trainers” module facilitates local adaptation and dissemination. Currently available as a field-testing version [80], the programme is being disseminated and evaluated in multiple countries to ensure contextual relevance across diverse health-care systems. The ICOPE Training Material (i.e., slide sets, facilitator guides, and guidance for the design and conduct of in-person training programmes) will likely be officially published in 2026, as the testing phase is over. Through this initiative, WHO aims to build a global health workforce capable of integrating ICOPE principles into routine primary care and promoting healthy ageing for all [81].

### AfriAGE—African Ageing and Geriatrics Network

Across Africa, capacity-building in ageing and geriatrics is supported by initiatives, such as IAGG-Africa, IAGG e-TRIGGER (AFMEE), and MENAAA. A project to establish an African Academy of Geriatrics (AAMEG) in 2020 was planned but had to be cancelled due to the COVID-19 pandemic [73]. The African Ageing and Geriatrics Network (AfriAGE) emerged from the Zimbabwe Geriatrics Network (Zim-GeN), originally founded through collaboration between the University of Zimbabwe and the University of Bristol in 2023 [82] to strengthen geriatric expertise in the region. As the initiative expanded, it recently evolved into AfriAGE, bringing together clinicians, nurses, researchers, and allied health professionals from Zimbabwe, South Africa, Uganda, Malawi, Zambia, and Botswana. The network, now with approximately 20 multidisciplinary members, meets quarterly for peer-to-peer teaching, alternating facilitators from African and United Kingdom institutions to support equitable knowledge exchange. In 2025, AfriAGE launched its first structured geriatric medicine online teaching programme, a weekly online course delivered via Zoom from March to May 2025, covering 10 major geriatric topics and attracting between 26 and 88 attendees per session [83]; this was provided free from charge, as AfriAGE was set up through research grant funding awarded to the University of Bristol in the United Kingdom.

## Discussion

This mapping reveals a dynamic and evolving landscape of transnational initiatives that collectively aim to advance education and training in geriatrics. The findings suggest the emergence of a tiered and interdependent global framework for geriatric education; however, this framework remains fragmented and unevenly connected. Its greatest potential lies in fostering complementarity by drawing on the diverse approaches and regional strengths of individual initiatives to create a more coherent, equitable, and sustainable global ecosystem for learning and development.

## Potential complementarities across regional and global initiatives

The mapping suggests complementarities amongst regional and global initiatives. At the global level, the IAGG, with its worldwide reach, regional divisions, and its foundational ICSO and e-TRIGGER programmes, appears well positioned to serve as a common partner for existing and emerging efforts in geriatric education and training. Within Europe, a framework with coordinating potential is suggested through the interactions of professional societies, accreditation bodies, and leadership academies. The EuGMS, together with initiatives such as PROGRAMMING CA21122, provides structures that support shared learning and opportunities for harmonisation. The UEMS-GMS contributes formal competency frameworks (ETR) and a knowledge-based examination (EGeMSE) that may serve as reference standards across borders. The EAMA strengthens clinical-academic leadership and mentorship, whilst the interdisciplinary orientation of EICA offers pathways to better connect research, education, and policy. Collectively, these mechanisms indicate a promising regional model of capacity for coherence and mutual reinforcement. A notable example of this is the joint endorsement of the 2025 UEMS-GMS ETR by EAMA, EuGMS, EICA, and IAGG [84].

In other regions of the world, several initiatives demonstrate potential to strengthen coherence and collaboration in geriatrics education. In the ASEAN region [85], the ACAI initiative (Tier 1) could further link with IAGG–Asia/Oceania (Tier 2) and, in turn, with the AAMA (Tier 3) to facilitate progressive advancement in specialist education and leadership across the region. In Africa, AfriAGE could be expanded through linkage with other Tier 1 initiatives, such as WHO ICOPE approach, e-TRIGGER AFMEE, and INIA, as well as with leadership academies such as MEAMA or future regional counterparts. In Australia and New Zealand, the VGMTP could align with relevant Tier 2 and 3 partners to strengthen leadership development and interregional collaboration. In Latin America and the Caribbean, PAHO provides a common framework for interaction, directly supporting the ACAPEM platform for primary care professionals, which contributes to building the foundations for the development of age-friendly services. This work is undertaken in collaboration with ALMA members, who are closely linked to PAHO leadership and include geriatricians prepared to transition into academic and leadership roles, as well as engagement with multidisciplinary teams and community health approaches. Strengthening linkages between regional capacity-building and interprofessional initiatives (Tiers 1 and 2) and leadership academies (Tier 3) could enhance vertical integration and mutual learning across regions. Ideally, regional initiatives would first map and connect activities within their own tier before establishing structured pathways to engage with programmes in other tiers, whether within their own region or through cross-regional collaboration. The effectiveness of a professional network depends largely on the frequency and quality of interactions amongst its members [86], highlighting the opportunity to strengthen existing initiatives through more deliberate interinstitutional collaborations.

At the global level, comparable potential exists for complementarity amongst initiatives, such as INIA, WHO ICOPE approach, and the IFA. Their respective mandates in education, policy, and capacity-building provide a strong basis for a connected global framework aligned with the goals of the UN Decade of Healthy Ageing (2021–2030) [6].

## Variable quality assurance and accreditation

Given persistent cross-country discrepancies in how geriatrics is recognised as a specialty and how training and standards are structured, both within Europe [87–89] and especially outside it [90], variability in quality and accreditation across transnational initiatives is expected. Moreover, some initiatives are explicitly designed to age-attune the general adult health-care workforce to geriatric principles and approaches, rather than (or in addition to) providing specialist training for geriatric practitioners, which further contributes to heterogeneity in educational objectives, standards, and accreditation frameworks.

Accreditation approaches may differ by tier and governance model. Tier 1 activities are frequently self-certified and issue provider certificates rather than profession-wide credentials, although some carry institutional endorsements and/or certificate of completion. Examples include IAGG’s e-TRIGGER under the FGE, WHO’s ICOPE training programme approach, UN’s INIA, and PAHO’s ACAPEM courses.

In Europe, a formal qualifications framework (EQF) exists [91]; however, transnational education and training initiatives in geriatrics are not currently mapped against it, largely because providers operate outside formal higher-education or vocational systems. Many Tier 2 medically relevant activities seek accreditation through the UEMS European Accreditation Council for Continuing Medical Education (EACCME), which provides an internationally recognised system for CME credit recognition. The EACCME has formal mutual recognition agreements with the American Medical Association (AMA) in the United States, the Royal College of Physicians and Surgeons of Canada (RCPSC), and the *Confederación Médica Latinoiberoamericana y del Caribe* (CONFEMEL) in Latin America and the Caribbean, allowing credits to be transferable across these systems [92]. Furthermore, the UEMS-GMS uses the ETR to articulate competency standards and, together with the EGeMSE, provides reference points that can support benchmarking and mobility, although neither constitutes a mandatory requirement across member states. Likewise, EAMA and its regional leadership academy counterparts award certificates of completion for their respective curricula, but these are not linked to formal examinations, professional training, or university accreditation within individual countries. Nevertheless, they carry high international esteem through informal peer recognition and sustained professional credibility within the global geriatrics education community.

In Australasia, the VGMTP aligns with the Royal Australasian College of Physicians (RACP) Geriatric Medicine curriculum and is supported by the Victorian health authorities and the Australian and New Zealand Society for Geriatric Medicine (ANZSGM), illustrating an endorsement pathway tied to a regional college.

This diversity enables responsiveness to local needs and fosters innovation, yet it also generates inconsistency and limits the portability of learning outcomes across borders. A tier-sensitive approach to quality assurance that links programmes to recognised frameworks could enhance coherence whilst maintaining flexibility. One potential anchor is the WHO Global Competency Framework for Universal Health Coverage (2022) [93], which provides an internationally endorsed structure outlining core competencies for health professionals across disciplines and levels of practice. Designed for adaptation across diverse contexts, it could serve as a reference for mapping geriatric education globally and regionally.

## Professional diversity

The mapping suggests that initiatives classified as Tier 3 tend to overrepresent the medical profession, whereas Tiers 1 and 2 are more consistently multidisciplinary in both design and participation. Addressing this imbalance and embedding interprofessional engagement across all tiers will be essential to achieving a more inclusive, equitable, and effective global framework for geriatrics education. Interprofessional inclusion remains critical to this advancement. Nurses, allied health therapists, pharmacists, psychologists, and social workers continue to be underrepresented in leadership roles and postgraduate education, despite their central contributions to the care of older persons. Future frameworks should therefore promote structured, team-based learning with equitable pathways for recognition, advancement, and leadership across all professional groups. European efforts in interprofessional education [94, 95], the work of PROGRAMMING CA21122 and other EU-funded research projects [96–99], and the EICA model, illustrate how interprofessional collaboration and leadership can be effectively embedded within educational systems. The growing emphasis on integration and interdisciplinarity in geriatrics education [100] highlights the value of these inclusive approaches and calls for their broader adoption at the global level.

## Barriers to participation: costs, language, digital access, and human resources

The mapping suggests a pattern in which registration fees rise from Tier 1 to Tier 3, ranging from free or low-cost online access (e.g., WHO’s ICOPE modules, PAHO’s ACAPEM courses, AfriAGE) to €12,500 for EAMA [101]. High tuition costs, travel requirements for in-person learning, limited institutional support, and variable industry sponsorship restrict participation, including from low- and middle-income countries. Long-term sustainability requires stable financing models, ownership, and diversified revenue strategies. Many initiatives rely on time-limited grants, volunteer faculty, or variable governmental support; without predictable funding streams, programmes struggle to scale or maintain continuity. Future frameworks should explore hybrid models for example combining governmental funding, professional society contributions, scholarship/bursary schemes, and partnerships to ensure continuity.

The predominance of English-language instruction further constrains engagement from non-English-speaking professionals and limits the local adaptation and dissemination of curricula. In addition, shortages of healthcare personnel, high clinical workloads, limited protected time for education, and constrained availability of trained faculty further restrict participation and delivery, particularly in under-resourced health systems where both learners and educators cannot be readily released for training. Digital transformation has created new opportunities to address some of these challenges. Programmes, such as ACAPEM, e-TRIGGER, and the WHO ICOPE approach, demonstrate the potential of online platforms to reach diverse audiences across continents, whilst ACAPEM, in particular, highlights how multilingual delivery can broaden participation. Nonetheless, persistent disparities in Internet connectivity, platform usability, and digital literacy continue to limit equitable access, especially in resource-constrained settings. To advance inclusion, future frameworks should adopt tier-linked strategies for equitable participation, offering open or subsidised entry for foundational programmes, fellowship schemes for mid-level training, and dedicated scholarships for Tier 3 academies. Expanding multilingual delivery, integrating translation technologies, and strengthening digital infrastructure partnerships with global and regional development agencies would further enhance affordability, accessibility, and participation. In addition, the proprietary nature of many training programmes, which are owned by individuals or institutions and carry intellectual property (IP) and commercial value, limits the sharing, adaptation, and contextualisation of educational materials and further restricts access for learners and institutions in resource-constrained settings.

## Need for global educational evaluation and research

Transparency and data sharing remain limited. Few transnational initiatives report essential parameters, such as target audience, content, delivery mode, certification or accreditation status, and costs. Establishing a coordinated global repository of educational programmes with an agreed taxonomy would enhance visibility, reduce duplication, and provide a foundation for comparative evaluation and evidence-based policy alignment. This repository could be developed under the auspices of WHO or IAGG, with shared governance to ensure inclusivity and continuous updating. Such a resource should be developed collaboratively, with multiple organisations curating shared and continuously updated content to ensure inclusivity and collective ownership. Digital platform requires ongoing maintenance, accreditation renewal, and content updates to remain aligned with evolving evidence and clinical standards. Establishing shared digital repositories, modular curricula, and open-source or low-cost platforms could reduce duplication and ensure longevity.

Evaluation of impact is also limited. Despite the availability of suitable evaluation frameworks [102, 103], a few programmes systematically assess learning outcomes, competency gains, or workforce and system effects, underscoring the need for coordinated educational evaluation and research to identify effective models and develop shared indicators of quality and impact. Innovative methods, such as microlearning, simulation, gamification, and peer-led learning, show potential to enhance engagement and retention but remain insufficiently validated. Strengthening the evidence base would not only enhance accountability but also build leadership capacity, inform investment, and support the integration of geriatrics education within broader health system and ageing policy frameworks.

## Regaining momentum in a post-pandemic world: the need for new generativity

The mapping indicates that the COVID-19 pandemic caused not only need for adaptations [104], but also disruption to educational continuity in geriatrics, halting residential academies such as SEAGM and delaying the establishment of new programmes, including the proposed African Academy of Geriatrics. These interruptions highlighted the dependence of many initiatives on in-person learning but also accelerated digital transformation, prompting rapid adoption of hybrid and virtual delivery formats. The pandemic also interrupted communication and collaboration amongst networks, slowing the exchange of knowledge and delaying the resumption of international training activities. At the same time, demographic changes within the professional community, particularly the retirement of senior educators and founding leaders, have underscored the need to invest in the next generation of academic and clinical leadership. Leadership academies can play an important role in this renewal by fostering continuity, strengthening mentorship, and supporting the development of strong and sustainable global and regional capacity in geriatrics education. As interest in longevity grows, there is a risk that attention and resources may become dispersed across a wide range of emerging ideas and approaches. Well-coordinated, evidence-based educational initiatives in geriatrics, grounded in high-quality research, can help ensure that focus remains aligned with validated, cutting-edge knowledge that promotes health span and quality of life in ageing populations.

## Limits of this current geriatric education inventory

This inventory has several limitations that should be acknowledged. First, it represents a descriptive, expert-informed mapping of selected transnational initiatives rather than a systematic review. Although some of the mapped initiatives are multilingual, the work was largely anchored in sources available in English, and national or subnational training programmes delivered in other languages are not described here. Second, many regional activities (e.g. those originating in USA and Canada), are likely to be underrepresented, reflecting both the eligibility criteria (focus on transnational rather than national or federal programmes) and incomplete information on regional initiatives at the time of data collection; future iterations of this work should explicitly invite additional contributions through open calls or other collaborative mechanisms. At the same time, representation and leadership from low- and middle-income countries remain uneven, highlighting an ongoing need to strengthen visibility, participation, and capacity-building in LMIC settings. Third, by design, topic-specific or subspecialty programmes (e.g. gerodontology, oncogeriatrics, orthogeriatrics, falls, frailty, dementia, and other focussed domains), as well as single-institution courses, were excluded, even though they represent important components of the broader educational ecosystem in ageing and geriatrics. Finally, the mapping provides a cross-sectional snapshot of initiatives between January and October 2025 and relies on public information and self-report from key informants; details on funding, sustainability, governance, and accreditation could not be independently verified, and no formal assessment of educational quality or outcomes was undertaken. These constraints mean that the present inventory should be interpreted as a pragmatic starting point for dialogue and coordination, rather than as a comprehensive or definitive catalogue of geriatric education worldwide.

Overall, a key limitation of the current geriatric education inventory is that many transnational initiatives focus primarily on introductory knowledge transfer and awareness raising. Whilst valuable, many of these activities do not support higher level competency development, skills acquisition, or measurable changes in clinical practice. This reflects a broader gap between informational training and transformative, competency-based education capable of improving care quality and health system performance.

## Conclusion

The global community faces a critical opportunity to build a coherent, equitable, and sustainable framework for education and training in geriatrics [3]. The initiatives mapped in this study, although diverse in their origins and scope, share a common goal of improving care for older people through professional education. Their collective potential reaffirms that excellence in geriatrics education can no longer be defined within regional or disciplinary boundaries but must be pursued as a global and collaborative endeavour [105]. Reducing duplication and achieving complementarity will require more than technical standardisation. It calls for a cultural shift from competition to cooperation, from fragmented programmes to coordinated systems, and from short-term projects to enduring partnerships. Equity, accessibility, and interprofessional inclusion must guide this transformation, so that all professionals, regardless of geography or discipline, can access learning opportunities and contribute to leadership in ageing care. The mapping indicates that future progress may depend less on the creation of new initiatives and more on the development of mechanisms for collaboration, mutual recognition, knowledge exchange, and wider dissemination. By fostering alignment and shared purpose, the global community can build an educational architecture that sustains progress across generations and ensures that both European and global geriatric medicine continue to advance and flourish [21].

## Supplementary Information

Below is the link to the electronic supplementary material.

Supplementary file1 (DOCX 615 kb)

## Data Availability

Not applicable.
