# Supplementary material for: Global education and training in geriatrics: mapping transnational initiatives and their complementarities
Source: Eur Geriatr Med. 2026 Feb 3;17(2):429–51. doi: 10.1007/s41999-026-01418-w (PMC13109137; doi:10.1007/s41999-026-01418-w)

**Supplementary Figure 1.** Some of the participants in the EuGMS Special Interest Group (SIG) on Education and Training session, “Global Education and Training Initiatives in Geriatrics,” held on 26 September 2025 during the 21st EuGMS Congress in Reykjavík, Iceland. The session brought together representatives from regional and international organisations to discuss complementarities and coordination across transnational initiatives in geriatric education and training.

Pictured on the screen (top to bottom): Matteo Cesari; José F. Parodi (left); Regina Roller-Wirnsberger (right); Luis Miguel Gutiérrez Robledo (left); Jugdeep Dhesi (right).

Pictured in the group (left to right): Fiona Ecarnot, Jūratė Macijauskienė, Liang-Kung Chen, Marina Kotsani, Tahir Masud, Karolina Piotrowicz, Manuel Montero-Odasso, M. Cristina Polidori, Mirko Petrovic, Maw Pin Tan, Román Romero-Ortuño, Adam L. Gordon, Nathalie van der Velde, Cornel Sieber, Michael Vassallo, Jean-Pierre Michel, Ashish Goel, Reshma A. Merchant, Prasert Assantachai, Finbarr C. Martin, Benedetta Bellotti.

Additional collaborators contributed to the work inside and outside this meeting.


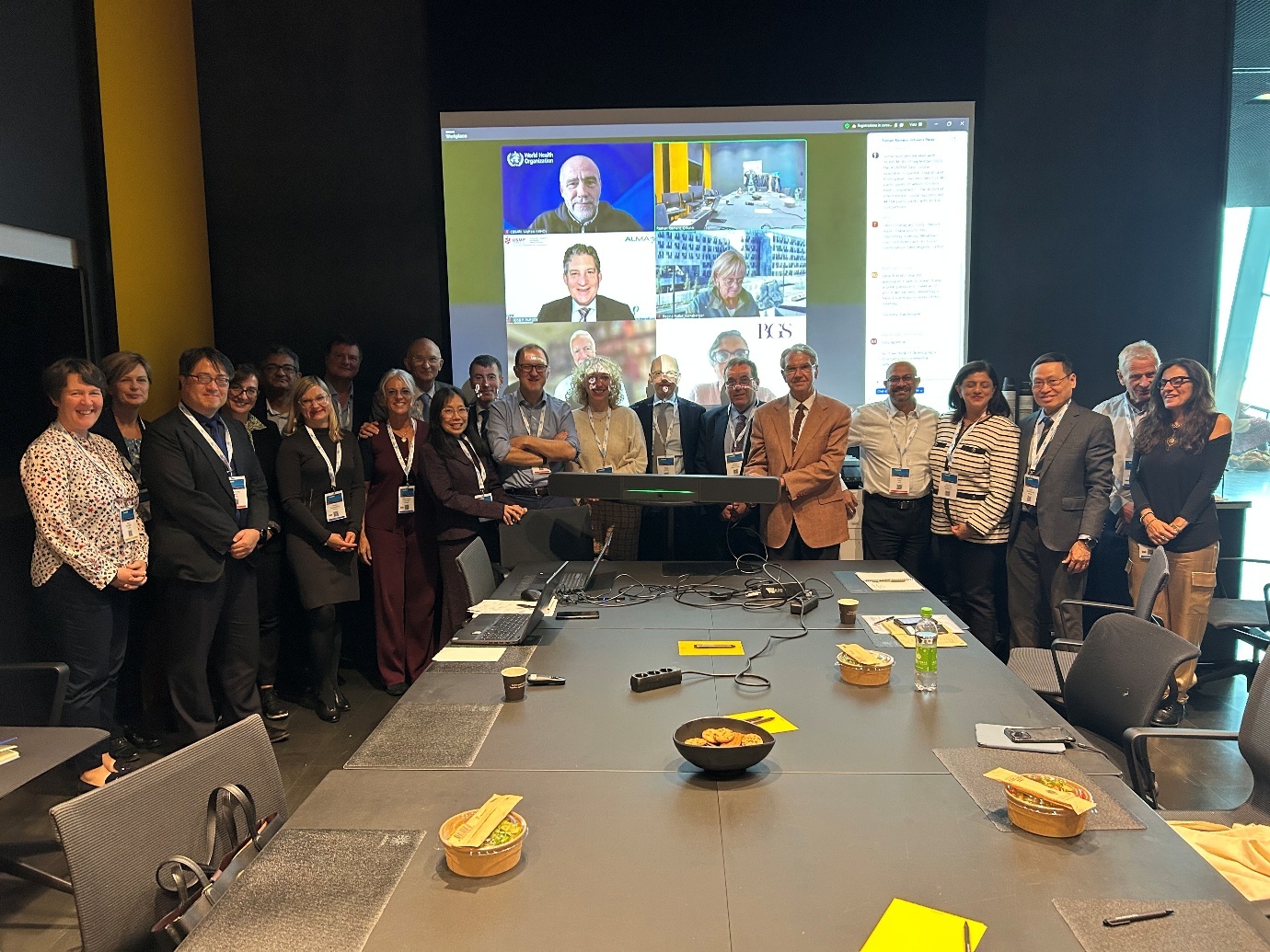

Supplement: Supplementary file 1 — Supplementary file1 (DOCX 615 kb) [file 41999_2026_1418_MOESM1_ESM.docx]
